# Integrated BSA-Seq and WGCNA Analyses Reveal Candidate Genes Associated with Winter Bud Dormancy Maintenance in Fruit Mulberry (*Morus* spp.)

**DOI:** 10.3390/cimb48010038

**Published:** 2025-12-27

**Authors:** Bing Sun, Zhaoxia Dong, Feng Zhang, Zhixian Zhu, Cheng Zhang, Cui Yu

**Affiliations:** 1 Institute of Economic Crops, Hubei Academy of Agricultural Sciences, Wuhan 430064, China; sunbio25@163.com (B.S.);; 2 Seedling Management Station of Hubei Provincial Forestry Bureau, Wuhan 430079, China

**Keywords:** *Morus* spp., dormancy, BSA sequence, WGCNA, fruit mulberry, candidate gene

## Abstract

The excessively concentrated ripening period of mulberries causes seasonal surplus. Fruit mulberry (*Morus* spp.) exhibits the unique trait of “simultaneous flowering and leaf flushing”, rendering budburst timing closely correlated with fruit ripening time. Thus, deciphering the molecular mechanism underlying winter bud dormancy maintenance in fruit mulberry is urgently needed. Herein, an F_1_ hybrid population comprising 337 individuals, derived from *Morus wittiorum* (♀) and ‘322’ (♂), was utilized as research material. Through Bulked Segregant Analysis Sequencing (BSA-Seq), we successfully mapped a dormancy-associated QTL interval designated as LB (Late Burst), spanning 9,990,001–11,990,000 bp on Chromosome 13. Integrating Weighted Gene Co-expression Network Analysis (WGCNA) results, *MaSVP* was identified as a candidate gene within this interval. Virus-induced gene silencing (VIGS) of *MaSVP* in winter buds of *Morus wittiorum* significantly accelerated budburst compared to the control, demonstrating that *MaSVP* represses winter bud dormancy release and plays a crucial role in regulating dormancy maintenance in fruit mulberry. Dynamic expression profiling of dormancy-related genes revealed that the transcript levels of *MaSVP*, *MaSAPK3*, *MaCASL2*, and *MaPYR8* were significantly downregulated (Tukey’s test, *p* < 0.05) as budburst approached, whereas those of *MaFT* and *MaGA20ox1-D* were significantly upregulated (Tukey’s test, *p* < 0.05). These results indicate that winter bud dormancy maintenance in *Morus wittiorum* is associated with abscisic acid (ABA) and gibberellin (GA) metabolism. Collectively, this study provides critical insights into the biological basis of winter bud dormancy maintenance in fruit mulberry and offers valuable genetic resources for breeding late-maturing cultivars.

## 1. Introduction

*Morus alba* L., a member of the genus *Morus* in the family Moraceae, is a perennial deciduous small tree or shrub [1]. Fruit mulberry (*Morus* spp.) is primarily cultivated for fruit production, with dual-purpose potential for both fruit and leaves [2,3]. Its ripe fruits, known as mulberries, possess high nutritional value [4], medicinal properties [5], and health-beneficial functions [6], and have been included in the list of “dual-purpose food-medicine list” [2,7]. Severe seasonal surplus and unsalable mulberries have emerged as a critical bottleneck, driven by the over-concentration of ripening periods across cultivars, synchronized market supply, and the inherent perishability of mulberries that limits their storage and transportation durability. A key contributing factor is the scarcity of early- and late-maturing varieties, with the majority of mainstream cultivars exhibiting concentrated ripening schedules. Mitigating this issue requires the rational integration of early-, mid-, and late-maturing fruit mulberry cultivars to maximize the extension of the fruiting and market supply period. The selection, breeding, and introduction of early- and late-maturing varieties not only optimize market supply dynamics but also enhance economic viability and meet consumer demand. Notably, mulberry growth initiates with the bud break of dormant winter buds, and the duration of winter bud dormancy is tightly correlated with the timing of mulberry ripening. Therefore, investigating the molecular mechanisms governing winter bud dormancy maintenance in fruit mulberries—with the aim of facilitating the breeding of early- and late-maturing cultivars—is scientifically pivotal and practically imperative [8]. This research will establish a critical theoretical foundation for overcoming the bottleneck of seasonal mulberry surplus, thereby advancing the sustainable development of the global fruit mulberry industry.

The timing of spring budburst in plants is directly associated with the duration of winter dormancy and the difficulty of dormancy release. Due to the periodic changes in environmental factors, plant growth and development exhibit a cyclic “growth–dormancy–growth” pattern. To adapt to adverse conditions or harsh winters, perennial plants undergo periodic dormancy to sustain their survival [9,10]. Based on more than 30 years of research, bud dormancy is classified into three types: para-dormancy, endo-dormancy (physiological dormancy), and eco-dormancy [11].

Endo-dormancy is triggered by the onset of autumn and winter, leading to a state of deep dormancy that requires induction by winter chilling temperatures and restoration of appropriate daylength for release [12]. In general, factors regulating plant dormancy include tree species and cultivars [13,14], external environmental cues (light, temperature, water) [15,16,17], internal factors (hormones, sugars, enzymes) [14,18,19], regulation by dormancy-associated genes [e.g., *PHY*, *CBF*, *CYC*, *DAM*, *SHORT VEGETATIVE PHASE* (*SVP*)] [20,21,22,23], and epigenetic regulatory mechanisms [24,25].

To date, relevant research has mainly concentrated on a restricted group of model species. Several genes regulating bud dormancy—such as those belonging to the SVP/AGL24 gene subfamily—have undergone more comprehensive studies across various taxa, and orthologs have been named *DAM* (*Rosaceae* fruit trees), *SVP2* (kiwifruit) (*Actinidia* spp.), and *SVL* (poplar), respectively [26]. Li et al. [27] identified six tandemly duplicated *DAM* genes in the peach (*Prunus persica*) mutant evergrowing (evg), where *PpDAM5* and *PpDAM6* exhibited down-regulated expression. Phylogenetic analysis revealed that *DAM* genes share the highest sequence similarity with *Arabidopsis thaliana SVP* and *AGL24*. In *Rosaceae* species, *DAM* genes are down-regulated in response to cold environments, triggering dormancy release [26], indicating their potential involvement in both dormancy induction and termination.

In transgenic poplar (*Populus* spp.) and apple (*Malus domestica*) over-expressing *PmDAM6* from Japanese apricot (*Prunus mume*), *DAM6* was up-regulated in leaf buds during dormancy establishment and down-regulated at budburst, accompanied by reduced growth phenotypes [28,29]. Silencing *MdDAM1* and *MdDAM4* in apple resulted in phenotypes analogous to the peach evg mutant [30]. *SVP* and *AGL24* are key regulators of the flowering pathway in *Arabidopsis thaliana*, modulating *FT* gene expression to influence flowering [20,31]. Notably, *FT-like* genes are associated with bud dormancy in temperate trees [31,32]. For example, the *Pyrus bretschneideri* (Chinese white pear) *PpDAM1* protein inhibits *PpFT2* expression, with the two genes showing reciprocal expression patterns during dormancy and dormancy release [33]. In kiwifruit [34] and grapevine (*Vitis vinifera*) [35,36], *SVP* acts as a flowering repressor by regulating the expression of *FT*, *SOC1*, and *FLC* [37].

In poplar, *SVL* overexpression induces dormant bud formation and inhibits bud break under short-day conditions via ABA mediation [38], potentially regulating ABA and gibberellin biosynthesis and signaling pathways [39]. In *Rosaceae* species, the molecular mechanisms through which *DAM* genes regulate dormancy have been partially clarified: *DAMs* may influence the ABA pathway in Japanese pear (*Pyrus pyrifolia*) [29]. In kiwifruit, *SVP2* overexpression alters the transcriptional levels of ABA and dehydration response pathways [40]. Chromatin immunoprecipitation sequencing (ChIP-seq) revealed that *SVP2* acts as a targeted negative regulator of plant growth-related genes, directly binding to those involved in the aforementioned pathways [40].

In this study, we implemented a robust mapping strategy to identify the major-effect quantitative trait loci (QTLs) governing winter bud dormancy maintenance in mulberry. An F_1_ mapping population, comprising 337 individuals, was generated from a cross between the late-maturing cultivar *Morus wittiorum* (maternal parent) and the early-maturing cultivar ‘322’ (paternal parent). Leveraging an integrated approach of BSA-seq and WGCNA, we aimed to: (i) delineate genomic intervals and major-effect QTLs associated with dormancy; (ii) prioritize candidate genes within these regions that facilitate dormancy maintenance; and (iii) elucidate the underlying molecular regulatory frameworks. These findings provide fundamental insights into the biological mechanisms of bud dormancy and offer critical theoretical support and germplasm resources for the molecular breeding of late-maturing mulberry varieties.

## 2. Materials and Methods

### 2.1. Plant Materials

#### 2.1.1. Materials for BSA Sequencing

An F_1_ hybrid population was constructed via sexual hybridization using *Morus wittiorum* as the maternal parent and ‘322’ as the paternal parent. After bud break induction in the laboratory, the seedlings were transplanted to the Ezhou Base of Hubei Academy of Agricultural Sciences, where all test plants received conventional field management. To capture the extreme variance in bud break timing, we rigorously characterized the F_1_ population and identified 30 individuals at each end of the phenotypic distribution. These individuals were subsequently utilized to construct two divergent bulks: early-breaking (E) and late-breaking (L). Young leaves were collected from each individual in the E and L bulks, along with leaves from the maternal parent (*Morus wittiorum*) and paternal parent (‘322’), resulting in a total of 62 samples for sequencing. Fresh young leaves were harvested from branches and immediately immersed in liquid nitrogen. Samples were preserved at −80 °C in an ultra-low temperature freezer until required.

#### 2.1.2. Materials for Expression Pattern Analysis

Winter buds of healthy *Morus wittiorum* plants were collected on 17 October 2022, 17 November 2022, 17 December 2022, 11 January 2023, 15 February 2023, 1 March 2023, 8 March 2023, 11 March 2023, 22 March 2023, and 28 March 2023. The plants were grown in the “Huazhong Branch of National Zhenjiang Mulberry Germplasm Repository and Hubei Mulberry Germplasm Repository” affiliated with the Industrial Crops Research Institute of Hubei Academy of Agricultural Sciences. Each sampling time point included three biological replicates. Collected buds were preserved at −80 °C in an ultra-low temperature freezer until required.

#### 2.1.3. Materials for VIGS Assay

On 15 February 2023, current-season shoots were harvested from vigorous 3-year-old *Morus wittiorum* individuals maintained at the aforementioned germplasm repository. The collected shoots were partitioned into segments, each retaining three to four winter buds, with their proximal ends submerged in distilled water for pre-acclimatization. The virus-induced gene silencing (VIGS) vectors, pTRV1 and pTRV2, were procured from Hainan Nixing Biotechnology Co., Ltd. (Haiko, Hainan, China). Agrobacterium tumefaciens strain GV3101 and Escherichia coli strain TOP10 were obtained from Shanghai Weidi Biotechnology Co., Ltd. (Shanghai, China).

### 2.2. DNA Library Construction and Sequencing

Genomic DNA (gDNA) was isolated from each sample using the CTAB method [41]. 1% agarose gel electrophoresis and a NanoDrop 2000 spectrophotometer (Thermo Scientific, Wilmington, DE, USA) were employed to verify the integrity and quality of the extracted gDNA. Equal volumes of gDNA from samples with the same phenotypic background were pooled to construct four libraries for BSA-seq: the early-breaking bulk (E), late-breaking bulk (L), maternal parent (*Morus wittiorum*, CSS), and paternal parent (‘322’). TruSeq DNA PCR-Free Prep Kit’s standard protocol was adopted for the preparation of sequencing libraries with a 400 bp insert size. After quality control and quantification, libraries with concentration ≥ 2 nM were qualified for PE sequencing (2 × 150 bp) on an Illumina NovaSeq platform (NGS technology).

### 2.3. BSA Analysis

Clean reads were mapped to the reference genome [2] using the mem algorithm of BWA (v0.7.12-r1039) with default parameters. Initial alignments were processed, sorted, and converted to BAM format via Picard (v1.107; http://www.psc.edu/index.php/user-resources/software/picard/ (accessed on 23 August 2022)). Genomic variants, including SNPs and InDels, were identified using GATK (4.1.8.1) [42] UnifiedGenotyper (v3.8) with a minimum confidence threshold for calling (stand_call_conf = 30) and emitting (stand_emit_conf = 10). To ensure a high-fidelity variant set, raw SNPs were subjected to rigorous hard-filtering based on the following criteria: FS ≤ 60.0, HaplotypeScore ≤ 13.0, MQ ≥ 40.0, QD ≥ 2.0, ReadPosRankSum ≥ −8.0, and MQRankSum > −12.5. Additionally, loci supported by fewer than four alternative reads or harboring missing genotypes were discarded to maintain dataset integrity. Functional annotation of the filtered variants was performed using ANNOVAR (2022Oct05) [43].

Candidate genomic regions associated with the target trait were identified using a BSA analysis pipeline (https://github.com/xiekunwhy/bsa/ (accessed on 28 August 2023)). The linkage strength between population-specific SNP/InDel loci and the target trait was quantified using the Euclidean Distance (ED) method [44,45]. Higher ED values indicate stronger linkage and tighter association between the SNP/InDel loci and the target trait. The parameters used for the sliding windows analysis are as follows: perl slidewindow.pl -i bsa/bsa.ed.xls -k bsa.ed -o bsa/ -f har.fa.fai -w 2000 -s 10 -cp 1,2 -cv 3 -ms 10.

### 2.4. WGCNA

The gene expression matrix (Table S1) was derived from unpublished RNA-seq data of *Morus wittiorum* (G14) and *Morus alba* (G15) generated previously by our research group. RNA-seq samples were collected from healthy G14 and G15 plants grown in the “Huazhong Branch of National Zhenjiang Mulberry Germplasm Repository and Hubei Mulberry Germplasm Repository” affiliated with the Industrial Crops Research Institute of Hubei Academy of Agricultural Sciences.

Winter buds of G14 were sampled on 26 January 2022 (G14_126), 16 February 2022 (G14_216), 10 March 2022 (G14_310), and 28 March 2022 (G14_328, initial bud break). For G15, winter buds were collected on 26 January 2022 (G15_126), 16 February 2022 (G15_216), 10 March 2022 (G15_310, initial bud break), and 15 March 2022 (G15_315, bud burst stage). RNA-seq analysis yielded an expression matrix encompassing 20,365 genes (Table S1).

Weighted gene co-expression networks were constructed using the WGCNA R package (v1.71) [46] based on FPKM-normalized expression profiles. Input data were log_2_(x+1) transformed and filtered to retain genes in the top 75% of Median Absolute Deviation (MAD > 0.01), followed by the removal of outliers and incomplete entries. To achieve scale-free topology, the soft-thresholding power (β) was selected at the first point where the model fit index (R^2^) reached 0.85. Co-expression modules were identified via dynamic tree cutting (deepSplit = 2, minModuleSize = 30) and subsequently merged based on a similarity threshold of >0.75 (MEDissThres = 0.25). Module-trait associations were quantified by Pearson correlation between module eigengenes (MEs) and phenotypic variables. Functional significance of module-specific genes was assessed through GO and KEGG pathway enrichment analyses using TBtools-II (version 2.312) [47] with default settings.

### 2.5. Gene Cloning and Vector Construction

Total RNA extraction and purification, first-strand cDNA synthesis, and recovery and purification of PCR amplicons were performed using the EASY spin Plus Plant RNA Kit, TRUEscript 1st Strand cDNA Synthesis Kit With gDNA Eraser, and Agarose Gel Purification Kit (all from Aidlab Biotechnologies, Beijing, China), respectively, following the manufacturers’ protocols. Nimble Cloning (NC) technology [48] was employed for target vector construction: target fragments were inserted into the destination vector using the Nimble Cloning Kit. High-fidelity PCR amplification was carried out with 2 × Phanta^®^ Max Master Mix (Vazyme Biotech, Nanjing, China) using first-strand cDNA as the template, following the kit instructions. The pNC-PCR products were subsequently cloned into the destination vector via NC reaction using the Nimble Cloning Kit, in accordance with the manufacturer’s protocol.

### 2.6. Bioinformatics Analysis of Target Genes

Candidate *MaSVP* genes were identified by querying the proteome with *Arabidopsis thaliana* SVP sequences using BLASTP (https://blast.ncbi.nlm.nih.gov/Blast.cgi?PROGRAM=blastp&PAGE_TYPE=BlastSearch&LINK_LOC=blasthome) (accessed on 28 November 2024), applying a stringent significance threshold of E-value < 1 × 10^−5^. To ensure structural integrity, conserved domains within the putative proteins were validated via the NCBI Conserved Domain Database (CDD, https://www.ncbi.nlm.nih.gov/Structure/cdd/wrpsb.cgi) (accessed on 28 November 2024) using an equivalent E-value < 1 × 10^−5^ cutoff. Multiple sequence alignments of the amino acid sequences were performed using BioEdit. Phylogenetic reconstruction was subsequently implemented in MEGA 7.0 using the Neighbor-Joining (NJ) algorithm. Nodal support was evaluated through bootstrap analysis with 1000 replicates.

### 2.7. VIGS-Mediated Gene Silencing

#### 2.7.1. Selection of VIGS Silencing Fragments and Vector Construction

Potential siRNA target sites within the *MaSVP* gene sequence were predicted using the online tools siDirect v.2.0 (sidirect2.rnai.jp/design.cgi) (accessed on 18 November 2022) and SGN-VIGS Tool (https://vigs.solgenomics.net/) (accessed on 18 November 2022). Fragments with high prediction scores were selected as VIGS fragments. Specific primers were designed to clone the selected fragments, which were then inserted into the pTRV2 vector. Sequencing-verified recombinant plasmid was extracted and purified prior to transformation into *Agrobacterium tumefaciens* strain GV3101. *Agrobacterium tumefaciens* strains harboring the target constructs or the empty pTRV2 vector (control) were cultured in LB medium supplemented with 50 mg/L kanamycin, 20 mg/L rifampicin, and 20 mM acetosyringone (AS). The cultures were incubated at 28 °C and 200 rpm in darkness until an OD_600_ of ~1.0 was reached. Cells were harvested via centrifugation, washed three times with sterile ddH_2_O, and resuspended to a final OD_600_ of 1.0 in an infiltration buffer (50 mM MES, 0.15 mM AS, 10 mM MgCl_2_, 0.02% Silwet L-77, and 0.5% (*v*/*v*) Tween-20) [49]. For infiltration, *Agrobacterium* containing pTRV1 was mixed in an equal volume (1:1, *v*/*v*) with those carrying pTRV2-target or pTRV2-empty. Robust winter buds at the basal end of excised *Morus wittiorum* branches were selected for syringe infiltration, with three independent biological replicates per treatment. Post-infiltration, the branches were maintained in distilled water to preserve vitality and transferred to a growth chamber at 20 °C and 70% relative humidity under a 12 h photoperiod for bud-break induction. At 7 days post-infiltration (dpi), phloem tissues from the distal (upper) bark were collected for genomic DNA extraction to minimize residual *Agrobacterium* contamination. Positive transformants were identified via PCR amplification using pNC-T specific primers (Table S3), followed by visualization on 1% agarose gels to confirm the presence of diagnostic amplicons.

#### 2.7.2. Screening of VIGS-Silenced Plants

Seven days after infiltration, genomic DNA was extracted from the treated branches for screening of positive silenced plants. PCR amplification was performed using the extracted DNA as the template and pNC-T-specific primers (Table S3). Following 1% agarose gel electrophoresis separation, specific bands in the PCR products confirmed positive silenced plants.

#### 2.7.3. Phenotypic and Relative Expression Analysis of VIGS-Silenced Plants

Detached branches infiltrated with the empty pTRV2-GFP vector served as the control group. The bud break time of VIGS-silenced plants was contrasted with that of control plants, and their phenotypes were documented through photography. Total RNA was isolated from both control and silenced plants, followed by reverse transcription for first-strand cDNA synthesis. qRT-PCR assays were conducted to measure the relative expression levels of target genes in VIGS-silenced plants.

### 2.8. Data Statistics and Analysis

The NCBI Primer Design Tool was used to design qRT-PCR primers (Table S2), and *MaActin* was chosen as the reference gene [8]. For target gene relative expression detection, qRT-PCR was conducted on a Bio-Rad Real-Time PCR Detection System (Bio-Rad Laboratories, Hercules, CA, USA) with SYBR^®^ Green Real-Time PCR Master Mix (Takara, Dalian, China) following the manufacturer’s protocol. Three biological replicates were set for all reactions, along with a negative control (sterile water without cDNA template), and the 2^−ΔΔCt^method [49] was employed to quantify gene relative expression. Data analysis and graph generation were performed using Origin Pro 2021 software (Origin Lab Corporation, Northampton, MA, USA).

## 3. Results

### 3.1. Budburst Trait and Population Construction of Fruit Mulberry

**Figure 1:**
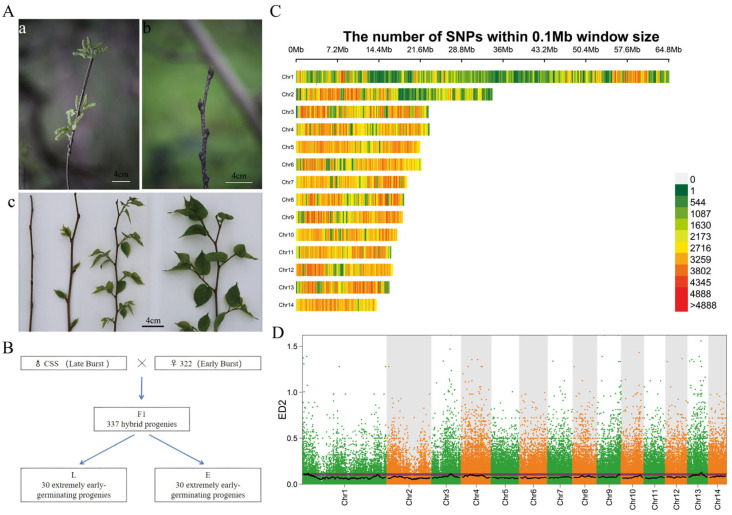
(**A**) Budburst phenotypes of the parental lines and F_1_ hybrid population. (**a**) The phenotypic photos of “322” (♂); (**b**) the phenotypic photos of *Morus wittiorum* (♀); (**c**) comparison of bud break in different individual plants of the F_1_ generation of “*Morus wittiorum*” × “322” population. The pictures were taken on 21 March 2022. (**B**) Schematic diagram of population construction and bulk pool sequencing. (**C**) Distribution of SNPs on chromosomes. (**D**). QTL localization results using ED methods. In the figure, the red line denotes a 0.99 confidence interval, while the blue line represents a 0.95 one. The red arrow points to the positioning interval under the 0.99 confidence interval.

In this study, *Morus wittiorum* (CSS, female parent, late-budding) and “322” (male parent, early-budding) were selected as parental lines, and an F_1_ (Figure 1A) segregating population was developed via artificial pollination. A genetic population consisting of 337 individuals was established, with all plants grown under conventional field conditions. Based on the budburst phenotyping of the F_1_ population conducted in early spring, two parental pools and two phenotypic bulks—an early-budding (E) bulk and a late-budding (L) bulk—were constructed (Figure 1B). All pooled DNA samples were sequenced on the Illumina NovaSeq platform (Figure 1A,B).

### 3.2. Identification of Candidate Intervals Regulating Winter Bud Dormancy in Mulberry

BSA-seq analysis yielded a total of 8,160,794 SNPs (Figure 1C, Table S4) and 1,236,220 InDels. Quantitative trait locus (QTL) mapping for late-budding trait in fruit mulberry was performed using the ED method (Figure 1D, Table S5). Integrating the mapping results of the two methods under the 0.99 confidence interval (marked by the red line in Figure 1D, Table S6), two QTL intervals were identified: one spanning 1,440,001–5,680,000 bp on Chromosome 1 (Chr1) and the other spanning 8,590,001–12,340,000 bp on Chr13. ED values of SNPs/InDels within 2000 Kb sliding windows were calculated (Table S7), with the top 10 windows listed in Table 1. The window spanning 9,990,001–11,990,000 bp on Chr13 exhibited the highest ED value (ED = 0.131). Thus, the candidate interval was narrowed down to 9,990,001–11,990,000 bp on Chr13, and this QTL was designated as LB (Late Budding).

### 3.3. GO and KEGG Enrichment Analyses of Mutant Genes in the Candidate Region

**Figure 2:**
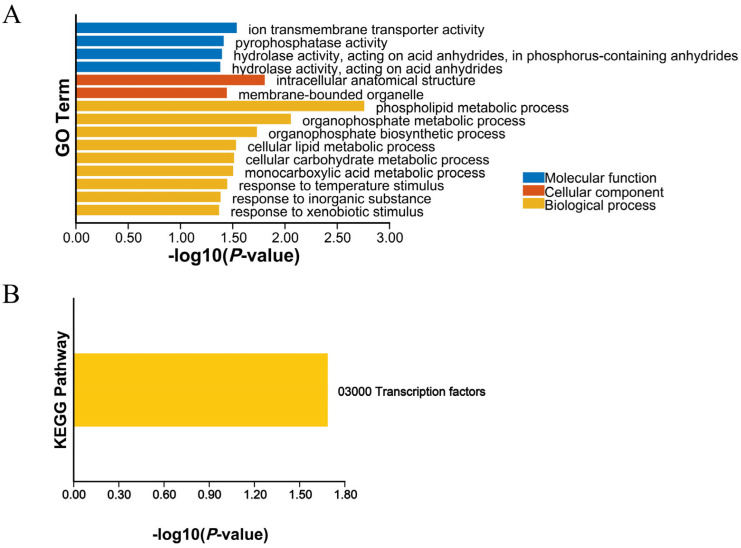
Enrichment analysis of GO (**A**) and KEGG (**B**) genes within the LB interval.

Based on the positional information of the LB interval, a total of 138 genes (M.alba_G0006266–M.alba_G0006403) were identified within this region. GO enrichment analysis of these interval genes (Figure 2A) revealed significant enrichment of the term “response to temperature stimulus” in the Biological Process category. This indicates that genes in the interval are involved in the regulation of temperature stimulus responses, a pathway potentially associated with plant dormancy. KEGG enrichment analysis (Figure 2B) revealed that interval genes were significantly enriched in the “Transcription Factors” pathway (7 genes), indicating that genes within this interval are involved in transcriptional regulation.

### 3.4. Selection of G14 and G15 Specific Modules

**Figure 3:**
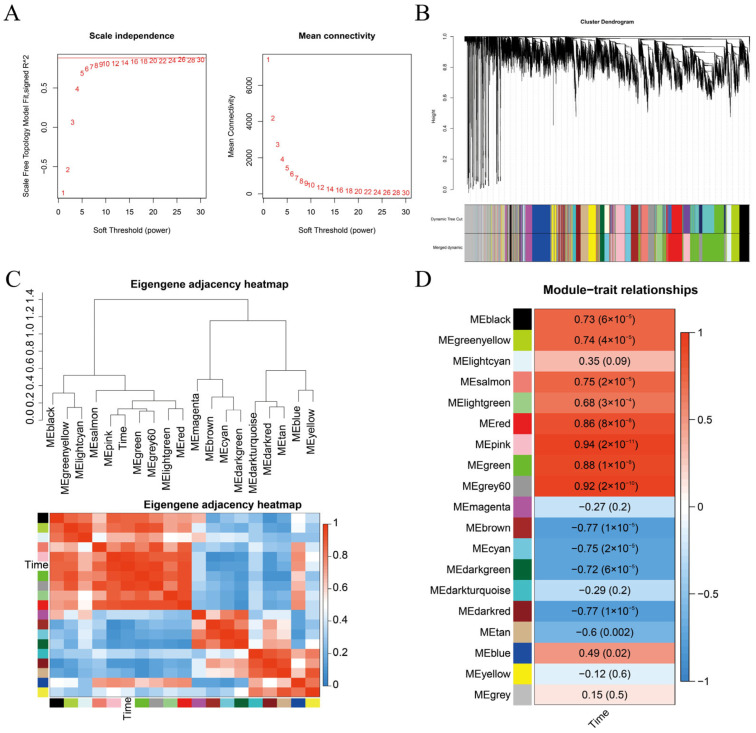
WGCNA of G14 and G15. (**A**) Soft threshold β Screening; (**B**) selection of gene co expression modules; (**C**) correlation heatmap analysis between phenotype and gene co expression modules; (**D**) selection of specific modules. In panel (**C**,**D**), red and blue intensities represent the strength of positive and negative correlations, respectively.

**Figure 4:**
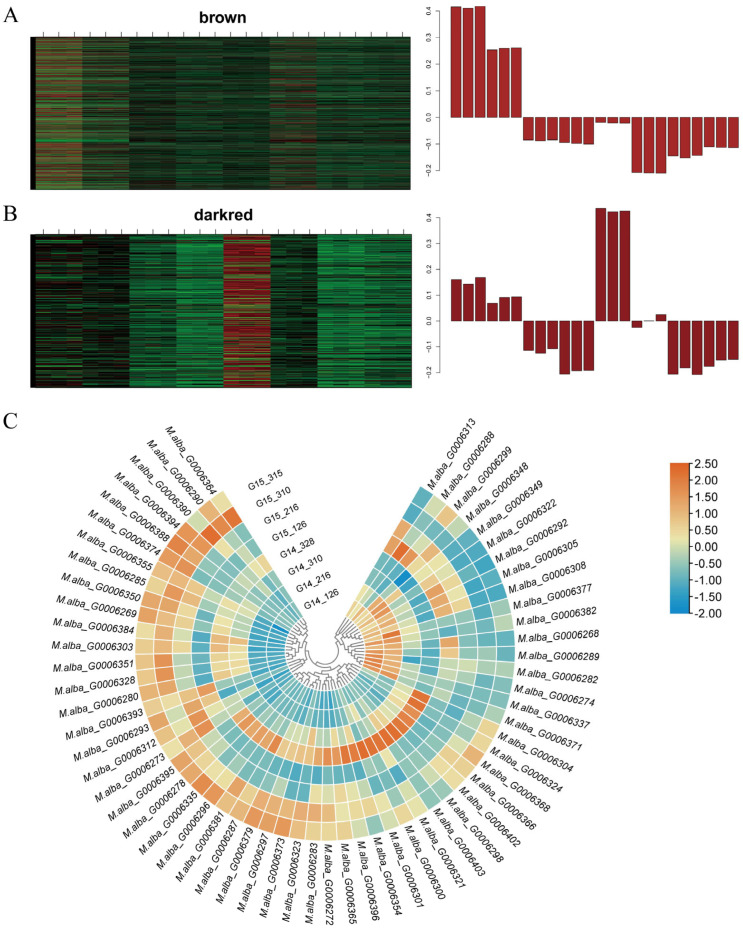
(**A**) Heat map analysis of gene expression patterns within brown modules. (**B**) Heat map analysis of gene expression patterns within darkred modules. In (**A**,**B**), increasing intensities of red and green signify upregulated and downregulated gene expression, respectively. (**C**) Clustering heatmap analysis of differentially expressed genes within the LB interval. In (**C**), increasing intensities of orange and blue signify upregulated and downregulated gene expression, respectively.

After data preprocessing of the expression matrix, 18,232 genes remained in the matrix. A soft-thresholding power (β) of 20 was selected (Figure 3A) to construct the weighted co-expression network, which ultimately yielded 19 distinct gene co-expression modules (Figure 3B). Correlation analysis between budburst time (time) and each module was conducted via WGCNA (Figure 3C). The correlation coefficients and *p*-values between each module and budburst time were calculated based on the time data (Figure 3D). Both the brown and darkred modules showed an extremely significant negative correlation with budburst time in fruit mulberry (−0.77, *p* ≤ 0.01). This indicates that genes within these two modules negatively regulate bud break in fruit mulberry (Figure 3D), serving as specific modules for maintaining winter bud dormancy. Heatmap analysis of genes within the two modules across different samples is presented in Figure 4A,B. Specifically, the majority of genes in the brown module (Figure 4A) and darkred module (Figure 4B) were highly expressed in dormant fruit mulberry buds before bud break and down-regulated as bud break approached. These modules are specific to dormancy maintenance and may play crucial roles in sustaining winter bud dormancy.

Heatmap analysis of genes within the two modules across different samples is presented in Figure 4A,B. Specifically, the majority of genes in the brown module (Figure 4A) and darkred module (Figure 4B) were highly expressed in dormant fruit mulberry buds before bud break and down-regulated as bud break approached. This indicates that the genes in these modules are highly associated with winter bud dormancy maintenance.

### 3.5. Selection of Candidate Genes

Analysis revealed 61 differentially expressed genes within the LB interval between winter buds of *Morus wittiorum* sampled at G14_126 and G14_328 (Figure 4C). We further analyzed the distribution of genes from the brown and darkred modules (dormancy maintenance-specific modules) within the LB interval (Table 2). Among these, 6 genes from the brown module and 1 gene from the darkred module were localized to the LB interval. Based on the gene significance (GS) values associated with budburst time (GS.time), *M.alba-G0006274* (designated as *MaSVP*) from the brown module was selected as the key candidate gene.

Differential expression analysis revealed that *M.alba_G0006274* (designated as *MaSVP*) exhibited a consistent down-regulation trend in winter buds of both *Morus wittiorum* and *Morus alba* from the G14_126 to G14_328 sampling stages (Figure 4C). Notably, the expression level of *MaSVP* in *Morus wittiorum* winter buds at G14_126 was higher than that in *Morus alba* at G15_126 (Figure 4C), which is consistent with the phenotypic trait that *Morus wittiorum* exhibits later budburst than *Morus alba*.

### 3.6. GO and KEGG Enrichment Analyses of MaSVP Co-Expressed Genes

**Figure 5:**
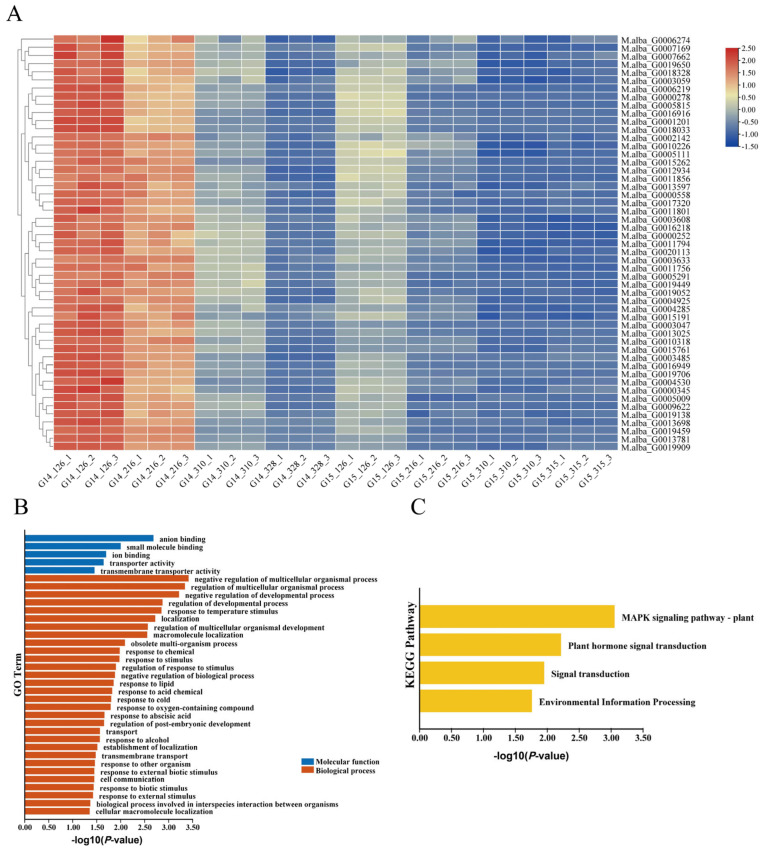
Heat map analysis (**A**), GO enrichment analysis (**B**) and KEGG enrichment analysis (**C**) of co expressed DEGs of *MaSVP* gene in brown module (t = 0.2).

Following the identification of *M.alba-G0006274* (designated as *MaSVP*) as a candidate gene (t = 0.2). Integrating differential expression results from transcriptomic data (Table S8), a total of 50 co-expressed differentially expressed genes (DEGs) were identified (Figure 5A, Table S9). After performing GO enrichment analysis on *MaSVP* and its 50 co-expressed DEGs (Figure 5B), the results showed that these genes were significantly enriched in GO terms related to the negative regulation of biological activities, including “negative regulation of metabolic process”, “negative regulation of cellular metabolic process”, “negative regulation of biological process”, etc. Additionally, enrichment was detected in the “response to abscisic acid (ABA)” term, which is related to ABA hormone metabolism—all of these processes are associated with plant dormancy.

A KEGG enrichment analysis of the co-expressed DEGs (Figure 5C) showed that a total of 4 co-expressed DEGs were significantly enriched in the “Plant hormone signal transduction” pathway. Notably, the ABA-related pathway within this category is associated with dormancy maintenance, which is consistent with the characteristic of late-budding genes that inhibit plant biological activities and promote dormancy. Collectively, *MaSVP* was confirmed as a key candidate gene for maintaining winter bud dormancy in fruit mulberry.

### 3.7. Expression Analysis of MaSVP and Other Dormancy-Associated Genes in the F_1_ Population

**Figure 6:**
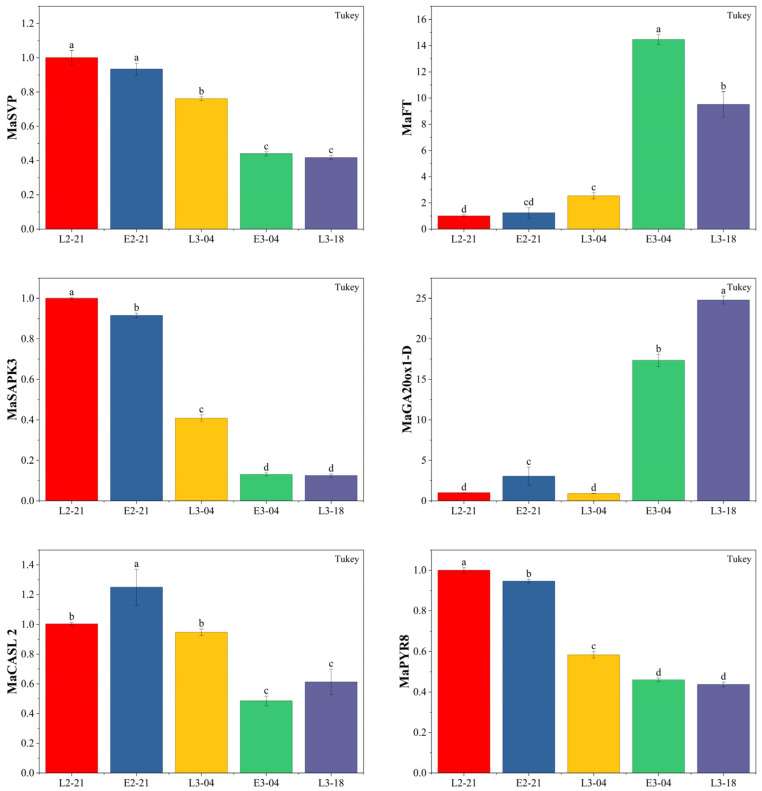
Expression dynamics of winter bud dormancy-associated genes in the F_1_ population at different developmental stages. L, late-budding extreme population; E, early-budding extreme population; L2-21, winter bud samples of the late-budding extreme population collected on 21 February (no sprouting); E2-21, winter bud samples of the early-budding extreme population collected on 21 February (no sprouting); L3-04, winter bud samples of the late-budding extreme population collected on 4 March (no sprouting); E3-04, winter bud samples of the early-budding extreme population collected on 4 March (initial bud break); L3-18, winter bud samples of the late-budding extreme population collected on 18 March (initial bud break). The same lowercase letters indicate no significant difference between treatments (*p* > 0.05) based on Tukey’s test.

To analyze the expression dynamics of dormancy-associated genes in the F_1_ population, six genes—*MaSVP*, *MaFT*, *MaSAPK3*, *MaGA20ox1-D*, *MaCASL2*, and *MaPYR8*—were selected for qRT-PCR analysis (Figure 6). *MaSVP* and *MaFT* are known regulators of plant bud break; *MaPYR8* and *MaSAPK3* are genes related to the ABA signaling pathway; *MaGA20ox1-D* encodes a key regulatory enzyme in the gibberellin (GA) biosynthesis pathway; and *MaCASL2* encodes a plant callose synthase. As bud break approached, the expression levels of *MaSVP*, *MaSAPK3*, *MaCASL2*, and *MaPYR8* genes significantly decreased (Tukey’s test, *p* < 0.05). Prior to bud break, their expression in the late-budding plants (L) was consistently higher than those in the early-budding plants (E) at the same sampling time points. In contrast, the expression of *MaFT* and *MaGA20ox1-D* was significantly up-regulated (Tukey’s test, *p* < 0.05) with the approaching bud break. Before bud break, their expression levels in L were significantly lower (Tukey’s test, *p* < 0.05) than those in E at the same sampling time points.

### 3.8. Cloning, Bioinformatics Analysis, and Functional Validation of MaSVP Gene in Fruit Mulberry

**Figure 7:**
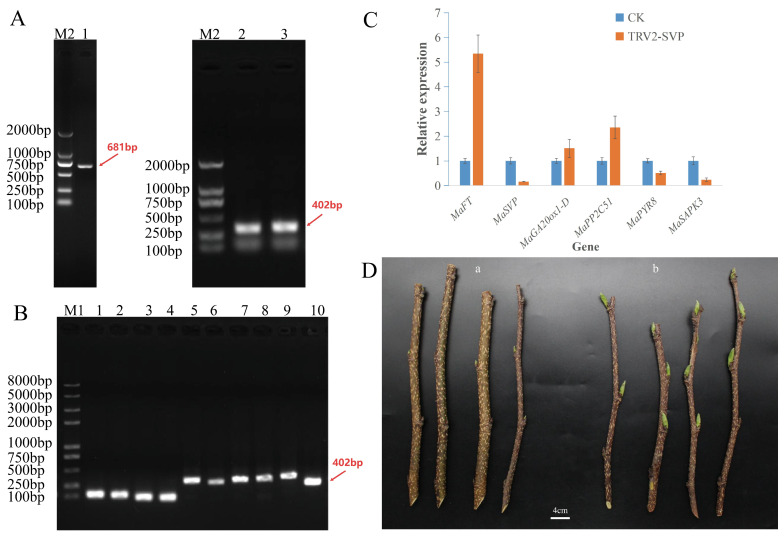
(**A**) *MaSVP* gene, VSVP fragment gel electrophoresis diagram. M2 is 2000 bp DNA marker, 1 is a *MaSVP* fragment; 3 and 4 are VSVP fragments. (**B**) Positive identification, M1 is 8000 bp DNA marker, 1–4 is positive control identification, 5–8 is positive pTRV2 SVP identification, 9 is positive Agrobacterium colony PCR identification, and 10 is *MaSVP* gene cDNA fragment inserted into the pTRV2 vector. (**C**) Analysis of gene expression levels related to CK group and TRV2-SVP group. (**D**) TRV-VIGS validation of *MaSVP* gene function. The comparison of bud break between CK group (**a**) and TRV2 SVP group (**b**) shows a ruler length of 4 cm in the figure.

Electrophoresis results showed that the PCR product of *MaSVP* was 681 bp in length (Figure 7A). The *MaSVP* gene contains a 681 bp open reading frame (ORF) encoding a protein of 226 amino acid residues with one stop codon. CD-search analysis (Figure S1) indicated that the protein encoded by *M.alba_G0006274* belongs to the Type II MADS-box protein family. A phylogenetic tree of *M.alba_G0006274* was constructed using MEGA 7 software (Figure S1). The results revealed that the protein encoded by *M.alba_G0006274* shares extremely high homology with the “*MADS-box protein JOINTLESS*” from *Morus notabilis*, and high similarity with the SVP protein from *Arabidopsis thaliana* and DAM proteins from *Malus domestica*. These findings suggest that *MaSVP* has functional similarity to these genes, encoding a DAM/SVP-like protein.

To analyze the dynamic expression pattern of *MaSVP* in winter buds of *Morus wittiorum* at different developmental stages (from 17 October, to 28 March), qRT-PCR analysis was performed (Figure S2). In the winter buds at each period, the gene expression level of *MaSVP* first increased significantly, reached the highest level on 11 January, then decreased significantly, and dropped to the lowest level on 28 March.

A 402 bp fragment of the *MaSVP* gene (Figure 7A) was selected as the gene silencing fragment (VSVP) to construct the pTRV2-VSVP silencing vector. Detached branches of *Morus wittiorum* were subjected to VIGS-mediated silencing via the injection method. Genomic DNA was extracted one week after treatment for positive identification by PCR (Figure 7B). Fifteen days post-treatment, winter buds of the control group were characterized by leaf greening; in contrast, TRV2-SVP-mediated silencing significantly advanced phenogeny, resulting in premature bud break (Figure 7D). Early budburst induced by *MaSVP* silencing indicated that *MaSVP* is involved in maintaining winter bud dormancy in fruit mulberry. qRT-PCR analysis of *MaSVP* expression in winter buds from the control (CK) and TRV2-SVP groups showed a significant downregulation of *MaSVP* in silenced buds (Figure 7C), confirming successful silencing at the mRNA level. Additionally, *MaSVP* silencing upregulated the expression of *MaFT*, *MaGA20ox1-D*, and *MaPP2C*, while decreasing the expression of *MaPYR8* and *MaSAPK3* in winter buds (Figure 7C).

## 4. Discussion

### 4.1. The Maintenance of Winter Bud Dormancy Is Critical for Breeding Fruit Mulberry Cultivars with Diverse Maturity Traits

The chilling requirement (CR) for breaking bud dormancy varies substantially among plant species and cultivars, and insufficient chilling accumulation frequently results in impaired bud break, reduced product quality, and compromised yield of horticultural crops—a phenomenon well-documented in apple (*Malus domestica*) [50]. Dormancy assessment is therefore indispensable for evaluating the relative CR of elite germplasm, ensuring consistent bud break and stable yield even under warm winter scenarios [51]. For perennial fruit trees, the duration of winter bud dormancy directly dictates flowering and fruiting phenology, thereby shaping cultivar maturity traits and economic value [29].

Fruit mulberry (*Morus alba*) exhibits a unique “simultaneous flowering and leaf flushing” trait, leading to a direct correlation between winter bud dormancy duration and fruit ripening time. This distinctive phenological characteristic underscores the significance of deciphering the molecular basis underlying winter bud dormancy maintenance in fruit mulberry. Uncovering key regulatory factors and pathways governing this trait will not only facilitate the breeding of late-maturing cultivars but also extend the market supply period of mulberry fruits, ultimately enhancing growers’ economic returns.

### 4.2. The Critical Regulatory Roles of QTL Interval LB and MaSVP in Maintaining Winter Bud Dormancy of Fruit Mulberry

Calle et al. [52] documented distinct amino acid mutations and structural variations in PavDAM proteins within the major QTL of an early-flowering sweet cherry (*Prunus avium*) F_2_ population, with these variations exhibiting strong co-segregation with low CR and early flowering traits. Notably, these mutations are conserved among early-flowering cultivars, suggesting that structural variations in *DAM* genes may underpin the development of low CR and early flowering phenotypes [29]. Herein, utilizing an F_1_ hybrid population of fruit mulberry, we employed BSA-Seq to map QTLs associated with winter bud dormancy maintenance (Figure 1), facilitating the identification of the candidate interval LB involved in regulating this trait (Figure 1D; Table 1). GO and KEGG enrichment analyses of the 138 genes within the LB interval (Figure 2) uncovered significant enrichment of “response to temperature stimulus” in the Biological Process category (Figure 2A). These findings indicate that genes within the LB interval mediate temperature stimulus responses—a pathway previously implicated in plant dormancy regulation across woody plants [53], highlighting the evolutionary conservation of temperature-associated dormancy regulatory networks.

Accumulating evidence corroborates the conserved repressive role of *DAM/SVP* family genes in bud dormancy. In transgenic poplar (*Populus trichocarpa*) and Japanese apricot (*Prunus mume*) overexpressing *DAM* genes, *DAM6* is up-regulated during dormancy establishment and down-regulated upon dormancy release [28,29]. Silencing *MdDAM1* and *MdDAM4* in apple leads to phenotypes analogous to the peach evg mutant, characterized by defective terminal and dormant bud formation [30], while suppressing *DAM* gene expression consistently induces early bud break [54]. In the present study, integrative analysis of WGCNA (Figure 3) and BSA-Seq pinpointed *MaSVP* as a candidate gene governing winter bud dormancy maintenance in fruit mulberry. The expression dynamics of *MaSVP*—across transcriptomic datasets (G14_126 to G14_328; Figure 4C), the F_1_ population (Figure 6), and winter buds of *Morus wittiorum* at distinct developmental stages (Figure S2)—consistently support its negative regulatory role in winter bud break. Critically, *MaSVP* silencing via VIGS resulted in accelerated winter budburst (Figure 7D), providing direct functional evidence for its regulatory function. Taken together, these findings establish that *MaSVP* is a key regulatory factor in maintaining winter bud dormancy of fruit mulberry, extending our understanding of the conserved yet species-specific roles of *SVP/DAM* family genes in woody plant dormancy.

### 4.3. Regulatory Mechanism of MaSVP in Maintaining Winter Bud Dormancy

SVP/DAM proteins are well recognized to orchestrate bud dormancy through integrating hormone signaling pathways, particularly the abscisic acid (ABA) pathway [33,39]. In plants, ABA binds to *PYR/PYL* receptors, inducing conformational changes that enable interaction with *PP2C* phosphatases and subsequent inhibition of their activity—a core step in ABA signal transduction [55]. For instance, *SVL* (a SVP homolog) activates *NCED3* expression to enhance ABA biosynthesis and upregulates *PYR* receptors, thereby reinforcing ABA signaling and promoting bud dormancy [39]. Conversely, *PP2Cs* act as negative regulators of ABA signaling: in pear (*Pyrus pyrifolia*), *PP2Cs* maintain low expression during dormancy and are up-regulated upon dormancy release, coinciding with decreased ABA levels [53]. Additionally, cross-talk between ABA and gibberellin (GA) signaling is critical for dormancy regulation—SVP/DAM proteins often repress GA biosynthesis genes (e.g., *GA20ox*) to reduce endogenous GA levels, further reinforcing dormancy [39,56].

In the present study, GO and KEGG enrichment analyses of *MaSVP* co-expressed DEGs revealed significant enrichment in “response to abscisic acid” and “Plant hormone signal transduction” pathways (Figure 5), indicating that *MaSVP* interacts with ABA metabolism-related genes to regulate winter bud dormancy. Functional validation via VIGS demonstrated that *MaSVP* suppression significantly increased the expression of *MaPP2C51* (a negative ABA signaling regulator) and decreased the expression of *MaPYR8* (ABA receptor) and *MaSAPK3* (a SnRK2 kinase in ABA signaling) (Figure 7C). These results align with the conserved role of SVP/DAM proteins in modulating ABA signaling, suggesting that *MaSVP* represses *PP2C* expression while enhancing ABA receptor and SnRK2 activity to reinforce ABA signaling during dormancy.

Beyond ABA signaling, SVP/DAM proteins also regulate dormancy by targeting flowering and GA biosynthesis genes. In white pear, *PpDAM1* represses *PpFT2* (a florigen gene) transcription by binding to its promoter, with *PpFT2* expression showing an inverse pattern to *PpDAM1* during dormancy and release [33]. Consistent with this, we observed a significant upregulation of *MaFT* following *MaSVP* silencing (Figure 7C), indicating that *MaSVP* inhibits *FT* expression to suppress bud break. In line with these reports, *MaSVP* silencing upregulated *MaGA20ox1-D* expression in winter buds (Figure 7C), suggesting that *MaSVP* restricts GA biosynthesis to maintain dormancy.

### 4.4. Summary and Future Perspectives

In summary, this study identified the QTL interval LB and *SVP/DAM* family gene *MaSVP* as key regulators of winter bud dormancy in fruit mulberry, and elucidated a regulatory mechanism wherein *MaSVP* modulates ABA-GA signaling cross-talk and *FT* expression to maintain dormancy. Three key contributions are highlighted: first, we mapped the first dormancy-associated QTL (LB) in fruit mulberry, providing a genetic framework for further dormancy-related gene mining; second, we functionally validated *MaSVP* as a core dormancy regulator, expanding the understanding of *SVP/DAM* gene function in Moraceae species; third, we uncovered a conserved yet species-specific regulatory pathway integrating ABA, GA, and flowering signals, shedding light on the evolutionary dynamics of dormancy mechanisms in woody plants.

Practically, the identification of *MaSVP* and the LB interval offers valuable genetic resources for marker-assisted selection (MAS) in late-maturing fruit mulberry breeding programs. Future studies could focus on: (1) identifying direct downstream targets of *MaSVP* through ChIP-Seq and yeast one-hybrid assays to clarify the detailed regulatory cascade; (2) investigating the interaction between *MaSVP* and environmental signals (e.g., temperature) to unravel how dormancy is modulated by external cues; (3) validating the application of *MaSVP* in MAS by evaluating its association with dormancy duration in natural fruit mulberry populations; and (4) exploring the potential of manipulating *MaSVP* expression to develop cultivars with tailored dormancy traits, adapting to changing climatic conditions.

## 5. Conclusions

In this study, an F_1_ hybrid population consisting of 337 individuals was developed via sexual hybridization between *Morus wittiorum* (female parent, elite late-maturing cultivar) and ‘322’ (male parent, early-maturing cultivar). Bulks of individuals with extreme phenotypes were constructed and subjected to fine mapping via BSA, together with the parental lines. Based on the comparison of ED values of SNPs/InDels within 2000 Kb sliding windows, a candidate interval spanning 9,990,001–11,990,000 bp on Chr13 was identified and designated as LB. Combined with WGCNA of RNA-Seq expression matrices from winter buds of *Morus wittiorum* (G14) and *Morus alba* (G15) at different developmental stages, *MaSVP* was uncovered as a candidate gene regulating winter bud dormancy within the LB interval. VIGS was used to silence *MaSVP*. Compared with the control, silencing of *MaSVP* resulted in early dormancy release of winter buds. Concomitant with *MaSVP* silencing, the expression levels of *MaGA20ox1-D* and *MaPP2C51* in winter buds were significantly upregulated (Tukey’s test, *p* < 0.05), while the expression of *MaPYR8* and *MaSAPK3* was significantly downregulated (Tukey’s test, *p* < 0.05). By elucidating the biological basis of winter bud dormancy maintenance in fruit mulberry, this study furnishes theoretical support and genetic resources for breeding late-maturing fruit mulberry cultivars.

## Figures and Tables

**Table 1:** Top 10 ED Values.

Chr	ED Method		
	Start (bp)	End (bp)	ED Value
Chr13	9,990,001	11,990,000	0.131076337
Chr13	9,970,001	11,970,000	0.130930557
Chr13	9,980,001	11,980,000	0.130924226
Chr13	9,960,001	11,960,000	0.130160023
Chr13	9,950,001	11,950,000	0.129617815
Chr13	9,910,001	11,910,000	0.129129828
Chr13	9,890,001	11,890,000	0.129018041
Chr13	10,000,001	12,000,000	0.128922806
Chr13	10,010,001	12,010,000	0.128922806
Chr13	9,900,001	11,900,000	0.128702361

**Table 2:** Differential expression gene information in brown and darkred module within the LB interval.

Gene_ID	Module Color	GS.Time	p.GS.Time	Annotation
M.alba-G0006274	brown	−0.836	3.64 × 10^−7^	AYK27567.1 short vegetative phase [*Morus alba var. alba*]
M.alba-G0006282	brown	−0.679	0.00026	XP_024028578.1 14-3-3-like protein GF_1_4 kappa [*Morus notabilis*]
M.alba-G0006337	brown	−0.775	8.55 × 10^−6^	XP_010087616.1 uncharacterized protein LOC21404965 [*Morus notabilis*]
M.alba-G0006355	brown	0.855	1.04 × 10^−7^	XP_010087591.1 BTB/POZ domain-containing protein DOT3 [*Morus notabilis*]
M.alba-G0006371	brown	−0.714	8.82 × 10^−5^	XP_010102575.1 uncharacterized protein LOC21387464 [*Morus notabilis*]
M.alba-G0006382	brown	−0.577	0.0032	XP_024017555.1 acyl-coenzyme A oxidase, peroxisomal [*Morus notabilis*]
M.alba_G0006299	darkred	0.355	0.0889	XP_010106297.1 E3 ubiquitin-protein ligase RING1 [*Morus notabilis*]

Note: GS.time is the correlation coefficient between genes and time, and p.GS.time is the *p*-value corresponding to this correlation coefficient.

## Data Availability

The original contributions presented in this study are included in the article/Supplementary Material. Further inquiries can be directed to the corresponding author(s).

## Supplementary Materials

The following supporting information can be downloaded at: https://www.mdpi.com/article/10.3390/cimb48010038/s1.

## Abbreviations

BSA-Seq	Bulked Segregant Analysis Sequencing
WGCNA	Weighted Gene Co-Expression Network Analysis
QTL	Quantitative Trait Loci
LB	Late Burst
VIGS	Virus-Induced Gene Silencing
SNPs	Single Nucleotide Polymorphisms
InDels	Insertions and Deletions
Chr	Chromosome
GO	Gene Ontology
KEGG	Kyoto Encyclopedia of Genes and Genomes
