# Supplementary figures and images for: Integrated BSA-Seq and WGCNA Analyses Reveal Candidate Genes Associated with Winter Bud Dormancy Maintenance in Fruit Mulberry (Morus spp.)
Source: Curr Issues Mol Biol. 2025 Dec 27;48(1):38. doi: 10.3390/cimb48010038 (PMC12840292; doi:10.3390/cimb48010038)

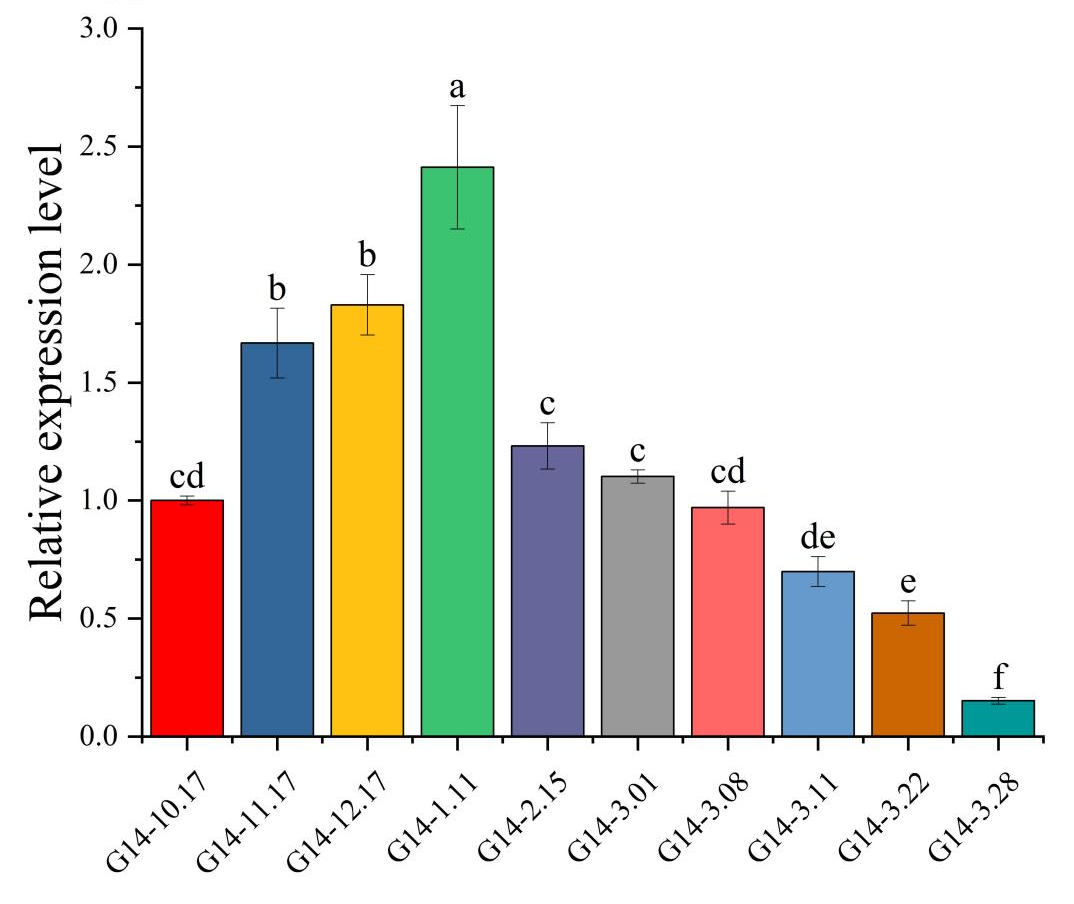

Supplement: Supplementary file 1 [file cimb-48-00038-s001.zip › Supplementary File(s)/Figure S2 The dynamic expression of MaSVP in winter buds of different periods.png]
